# Supplementary figures and images for: Conditional gene expression reveals stage‐specific functions of the unfolded protein response in the Ustilago maydis–maize pathosystem
Source: Mol Plant Pathol. 2019 Dec 3;21(2):258–71. doi: 10.1111/mpp.12893 (PMC6988420; doi:10.1111/mpp.12893)

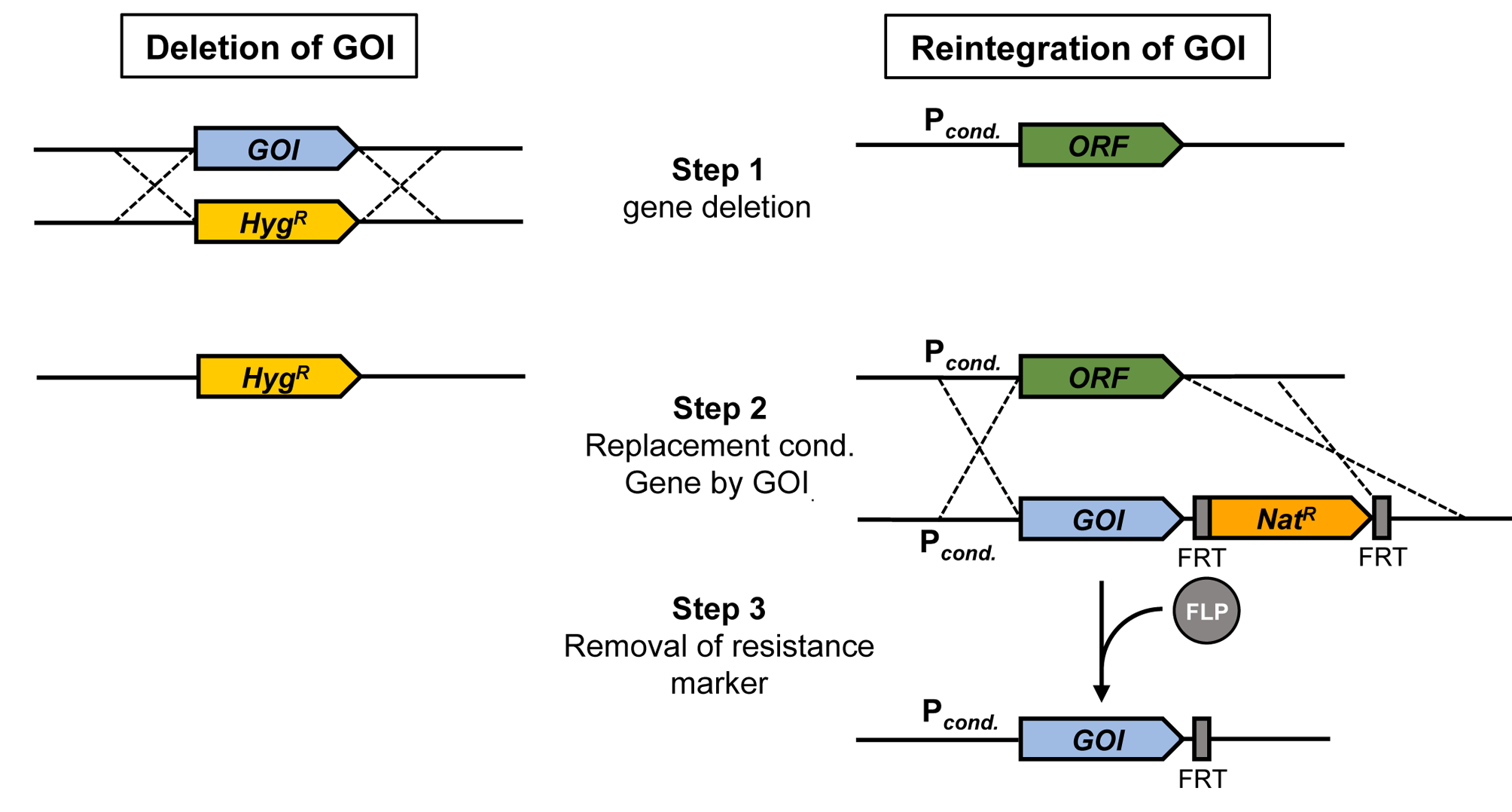

Supplement: Supplementary file 1 — Fig. S1 Strategy for strain generation for conditional gene expression. (1) The gene of interest (GOI) is deleted from its native genomic locus. (2) The GOI is integrated into the genomic locus of the conditionally expressed gene, thereby replacing the native gene. (3) The resistance marker (here, NatR) is removed using the FLP/FRT recombination system. [file MPP-21-258-s001.tif]

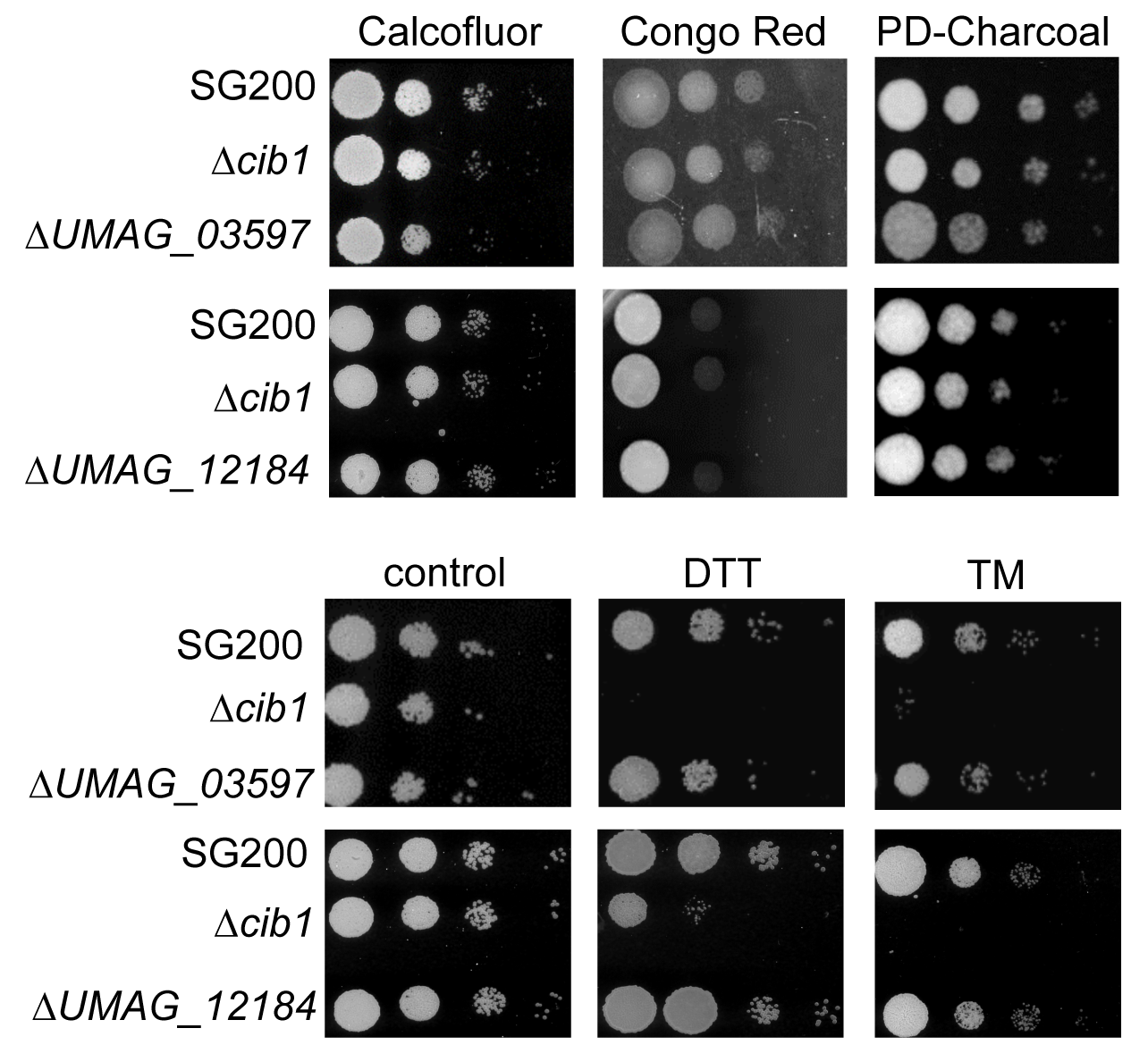

Supplement: Supplementary file 2 — Fig. S2 ∆UMAG_12184 and ∆UMAG_03597 strains do not show increased sensitivity to cell wall or endoplasmic reticulum (ER) stresses. Cell wall and ER stress assays, and tests for filamentous growth of strains SG200, SG200∆cib1, SG200∆UMAG_12184 and SG200∆UMAG_03597. Serial 10‐fold dilutions were spotted on YNB‐Glucose solid medium supplemented with Congo Red (100 µg/mL) or Calcofluor White (50 µM) to induce cell wall stress, and on YNBG solid medium supplemented with tunicamycin (TM) (1 µg/mL) or dithiothreitol (DTT) (1 mM) to induce ER stress. Cells were spotted on charcoal‐containing potato dextrose solid medium to induce filamentous growth. Pictures were taken after 48 h of incubation at 28 °C. [file MPP-21-258-s002.tif]
