# Supplementary material for: Conditional gene expression reveals stage‐specific functions of the unfolded protein response in the Ustilago maydis–maize pathosystem
Source: Mol Plant Pathol. 2019 Dec 3;21(2):258–71. doi: 10.1111/mpp.12893 (PMC6988420; doi:10.1111/mpp.12893)
Supplement: Supplementary file 3 — Table S1 Strains used in this study [file MPP-21-258-s003.docx]

**Supplemental Table S1: Strains used in this study**

| Strain | Relevant Genotype | Reference |
| --- | --- | --- |
| FB1 | *a1 b1* | Banuett and Herskowitz, 1989 |
| FB2 | *a2 b2* | Banuett and Herskowitz, 1989 |
| SG200 | *a1 mfa2 bE1bW2* | Kämper *et al.,* 2006 |
| FB1∆*cib1* | *a1 b1* ∆*cib1* | Heimel *et al.*, 2010 |
| FB2∆*cib1* | *a2 b2* ∆*cib1* | Heimel *et al.*, 2010 |
| UMH168 | *a1mfa2 bE1bW2* ∆*pit1/2* *ip^r^* P*_pit1/2_:pit1/2 ip^s^* | Hampel, 2016 |
| UMH172 | *a1mfa2 bE1bW2* ∆*pit1/2:pit1/2* (Nat^R^) | Hampel, 2016 |
| UMH247 | *a1mfa2 bE1bW2* ∆*pit1/2::pit1/2* (Hyg^R^) | Hampel, 2016 |
| UMH268 | *a1mfa2 bE1bW2* ∆*pit1/2::pit1/2* (-Hyg^R^) | Hampel 2016 |
| SG200∆*UMAG_12184* | *a1mfa2 bE1bW2* ∆*UMAG_12184* | This study |
| SG200∆*UMAG_03597* | *a1mfa2 bE1bW2* ∆*UMAG_03597* | This study |
| ULS62 | *a1 mfa2 bE1bW2 ip^r^* P*_mig2_1_:cib1^s^ ip^s^* | This study |
| ULS251 | *a1 b1* ∆*cib1* ∆*mig2_1::cib1^s^* (+*Nat^R^*) | This study |
| ULS264 | *a1 b1* ∆*cib1* ∆*mig1::cib1^s^ (-Nat^R^)* | This study |
| ULS270 | *a1 b1* ∆*cib1* ∆*mig2_1::cib1^s^ (-Nat^R^*) | This study |
| ULS282 | *a2 b2* ∆*cib1* ∆*UMAG_12184::cib1* | This study |
| ULS299 | *a2 b2* ∆*cib1* ∆*UMAG_03597::cib1* | This study |
| ULS352 | *a1 mfa2 bE1bW2 ip^r^ P_mig2_1_:cib1^s^ ip^s^* | This study |
