# Supplementary material for: Conditional gene expression reveals stage‐specific functions of the unfolded protein response in the Ustilago maydis–maize pathosystem
Source: Mol Plant Pathol. 2019 Dec 3;21(2):258–71. doi: 10.1111/mpp.12893 (PMC6988420; doi:10.1111/mpp.12893)
Supplement: Supplementary file 4 — Table S2 Primers used in this study [file MPP-21-258-s004.docx]

**Supplemental Table S2: Primers used in this study**

| **Purpose** | **Name** | **Sequence** | **Reference** |
| --- | --- | --- | --- |
| **qRT-PCR** | RT_cib1_rev | CATCGACGTTGTTTCCGGCCT | Heimel *et al.*, 2010a |
|  | RT_cib1_spliced_f | GCCTCCCTGCAGCGGATGC | Heimel *et al.*, 2010a |
|  | RT_eIF2b_f | ATCCCGAACAGCCCAAAC | Heimel *et al.*, 2010a |
|  | RT_eIF2b_r | ATCGTCAACCGCAACCAC | Heimel *et al.*, 2010a |
|  | RT_pr1_f | ACTACGTGGACCCGCACAAC | Doehlemann *et al.*, 2008 |
|  | RT_pr1_r | CGGAGTGGATCAGCTTGCAGTC | Doehlemann *et al.*, 2008 |
|  | RT_pr3_f | GAACAACTACAGCAGCCAGGTG | Doehlemann *et al.*, 2008 |
|  | RT_pr3_r | GAGACAATAGCTGACATGCGTC | Doehlemann *et al.*, 2008 |
|  | RT_pr5_f | TATCGGCCGGAATAGGCTCTG | Doehlemann *et al.*, 2008 |
|  | RT_pr5_r | CGCGTACATACAAATGCGTGC | Doehlemann *et al.*, 2008 |
|  | Pit1-RT-fw | GCCCAGTCCCAGTCTATC | Doehlemann *et al.*, 2008, modified |
|  | Pit1-RT-rev | GAAGGGGAGCAGGAGATG | Doehlemann *et al.*, 2011, modified |
|  | Pit2-RT-fw | CAAGAATCCGCCTGCCAACA | Doehlemann *et al.*, 2011, modified |
|  | Pit2-RT-rev | AGGATCTGTCGGCATGACC | Doehlemann *et al.*, 2011, modified |
| ***UMAG_12184* deletion** | um12184_LB_f | CCAAGGCTAACGTGATGGATAC | This study |
|  | um12184_LB_r | TATAGGCCATCTAGGCCGGTAACTTTCTGTCTTGTACAAGGT | This study |
|  | um12184_RB_f | TATAGGCCTGAGTGGCCAGCAGAGTAGGAACGAGTGGT | This study |
|  | um12184_RB_r | GACTCACAGACTCGACTGTG | This study |
| ***UMAG_03597* deletion** | um03597_KO_lr | TATAGGCCATCTAGGCCGGTGCAGAGCTGACGGAAG | This study |
|  | um03597_KO_lf | GGTGCCAGAGAGGATGCAAG | This study |
|  | um03597_KO_rf | TATAGGCCTGAGTGGCCCGTCAAGTCAACTTTACCGGTC | This study |
|  | um03597_KO_rr | GACGGTAACAACGCGTATACC | This study |
| **promoter fusions** | um12184_lr_SfiI | TATAGGCCACTCAGGCCGGTAACTTTCTGTCTTGTACAAGGT | This study |
|  | um12184_rf_BamHI | TATAGGATCCAGCAGAGTAGGAACGAGTGGT | This study |
|  | um03597_lr_SfiI | TATAGGCCACTCAGGCCGGTGCAGAGCTGACGGAAG | This study |
|  | um03597_rf_KpnI | TATAGGTACCCGTCAAGTCAACTTTACCGGTC | This study |
|  | mig1_LB_f | CCGTCAACTGCGCCAAGTG | This study |
|  | mig1_LB_r_SfiI | TATAGGCCACTCAGGCCCTTGATCTGGAGGAAGAGAATGG | This study |
|  | mig1_RB_f_BamHI | TATAGGATCCAGGCTGCTACAAGATCACGCT | This study |
|  | mig1_RB_r | GACGCAACAGGATGTCTTGTC | This study |
|  | mig2_1_LB_f | CTGCACAGCGGATGGCAAAG | This study |
|  | mig2_1_LB_r_SfiI | TATAGGCCACTCAGGCCCTTCTCTTGGCACGTGTTTTGG | This study |
|  | mig2_1_RB_f_BamHI | TATAGGATCCAAGACTAGCGTTGCTGCGTAG | This study |
|  | mig2_1_RB_r | CAGACTGAGAATGTCGATGTGG | This study |
|  | NatR_f_SfiI_frt | TATAGGCCTAGATGGCCAGAAGTTCCTATTCTCGAGAAAGTATAGGAACTTCAATTGCGGCCGCACCTAGAAG | This study |
|  | NatR_r_frt_BamHI | TATAGGATCCAGAAGTTCCTATACTTTCTCGAGAATAGGAACTTCGCGGCCGCACTCCTACA | This study |
|  | Hyg_f | TATAGGCCTAGATGGCCAGAAGTTC | This study |
|  | Hyg_r_KpnI | TATAGGTACCGAAGTTCCTATACTTTCTAGAGAATAG | This study |
|  | cib1s_SfiI_f | TATAGGCCTGAGTGGCCATGACTAGCACCACCACGTCA | This study |
|  | cib1s_SfiI_r | TATAGGCCATCTAGGCCTCAAGCGACGATTGAGGCCAT | This study |
