# Supplementary material for: Conditional gene expression reveals stage‐specific functions of the unfolded protein response in the Ustilago maydis–maize pathosystem
Source: Mol Plant Pathol. 2019 Dec 3;21(2):258–71. doi: 10.1111/mpp.12893 (PMC6988420; doi:10.1111/mpp.12893)
Supplement: Supplementary file 5 — Table S3 Plasmid used in this study [file MPP-21-258-s005.docx]

**Supplemental Table S3: Plasmid used in this study**

| **Name** | **Full name** | **Locus** | **Insert** | **Resistance (*E. coli*)** | **Resistance (*U. maydis*)** |
| --- | --- | --- | --- | --- | --- |
| pLS25 | pJet1.2 mig1-FL #5 | *mig1* | *GFP* | ampicillin | hygromycin |
| pLS27 | pJet1.2 mig2_1-FL #2 | *mig2_1* | *GFP* | ampicillin | hygromycin |
| pLS29 | pJet1.2 mig2_2-FL #1 | *mig2_2* | *GFP* | ampicillin | hygromycin |
| pLS31 | pJet1.2 mig2_3-FL #6 | *mig2_3* | *GFP* | ampicillin | hygromycin |
| pLS35 | pCR2.1 pum05690:mCherry #1 | *UMAG_05690* | *mCherry* | ampicillin | hygromycin |
| pLS36 | pCR2.1 pum12184:mCherry #1 | *UMAG_12184* | *mCherry* | ampicillin | hygromycin |
| pLS37 | pCR2.1 pum03597:mCherry #3 | *UMAG_03597* | *mCherry* | ampicillin | hygromycin |
| pLS34 | pCR2.1 pum00050:mCherry FL | *UMAG_00050* | *mCherry* | ampicillin | nourseothricin |
| pLS45 | pJet1.2 pmig1:cib1s #4 (NatR) | *mig1* | *cib1(spliced)* | ampicillin | nourseothricin |
| pLS46 | pJet1.2 pmig2_1:cib1 #5 (NatR) | *mig2_1* | *cib1* | ampicillin | nourseothricin |
| pLS56 | pJet1.2 pmig2_2:cib1 #3 (NatR) | *mig2_2* | *cib1* | ampicillin | nourseothricin |
| pLS52 | pCR2.1 pum12184:cib1 #3 (NatR) | *UMAG_12184* | *cib1* | ampicillin | nourseothricin |
| pLS60 | pCR2.1 pum03597:cib1 #1 (NatR) | *UMAG_03597* | *cib1* | ampicillin | nourseothricin |
